# Deficiency of C5L2 Increases Macrophage Infiltration and Alters Adipose Tissue Function in Mice

**DOI:** 10.1371/journal.pone.0060795

**Published:** 2013-04-22

**Authors:** Danny Gauvreau, Abhishek Gupta, Alexandre Fisette, Fun-Qun Tom, Katherine Cianflone

**Affiliations:** 1 Institut Universitaire de Cardiologie et de Pneumologie de Québec Research Center (CRIUCPQ), Laval University, Quebec, Quebec, Canada; 2 Department Medicine, Faculty of Medicine, Laval University, Quebec, Quebec, Canada; Wageningen University, Netherlands

## Abstract

**Background:**

Obesity is considered as a systemic chronic low grade inflammation characterized by increased serum pro-inflammatory proteins and accumulation of macrophages within white adipose tissue (WAT) of obese patients. C5L2, a 7-transmembrane receptor, serves a dual function, binding the lipogenic hormone acylation stimulating protein (ASP), and C5a, involved in innate immunity.

**Aim:**

We evaluated the impact of C5L2 on macrophage infiltration in WAT of wildtype (Ctl) and C5L2 knock-out (C5L2^−/−^) mice over 6, 12 and 24 weeks on a chow diet and moderate diet-induced obesity (DIO) conditions.

**Results:**

In Ctl mice, WAT C5L2 and C5a receptor mRNA increased (up to 10-fold) both over time and with DIO. By contrast, in C5L2^−/−^, there was no change in C5aR in WAT. C5L2^−/−^ mice displayed higher macrophage content in WAT, varying by time, fat depot and diet, associated with altered systemic and WAT cytokine patterns compared to Ctl mice. However, in all cases, the M1 (pro-) vs M2 (anti-inflammatory) macrophage proportion was unchanged but C5L2^−/−^ adipose tissue secretome appeared to be more chemoattractant. Moreover, C5L2^−/−^ mice have increased food intake, increased WAT, and altered WAT lipid gene expression, which is reflected systemically. Furthermore, C5L2^−/−^ mice have altered glucose/insulin metabolism, adiponectin and insulin signalling gene expression in WAT, which could contribute to development of insulin resistance.

**Conclusion:**

Disruption of C5L2 increases macrophage presence in WAT, contributing to obesity-associated pathologies, and further supports a dual role of complement in WAT. Understanding this effect of the complement system pathway could contribute to targeting treatment of obesity and its comorbidities.

## Introduction

Obesity is associated with ‘metaflammation’, characterized by a systemic chronic low grade inflammation, differing from classic acute inflammation [1], [2]. Metaflammation involves the traditional inflammatory response to pathogens including systemic increases of C reactive protein (CRP) [3] and interleukin 6 (IL-6) [4], recruitment of leukocytes to the site of inflammation, activation of resident leukocytes and initiation of reparative tissue responses [5]. Nevertheless, metaflammation differs by its multi-organ target and chronic low-level activation of the innate immune response, as seen in obesity, which affects metabolic homeostasis over time [2]. This inflammation is assumed to lead to insulin resistance which could ultimately induce type 2 diabetes [6] and is considered a precursor for other obesity-associated pathologies.

The obesity inflammatory response is triggered by and located predominantly in adipose tissue [7], a well known energy reserve and endocrine organ, secreting multiple adipokines, that contribute to systemic cytokine levels. At a tissue level, adipose tissue inflammation in obese subjects is directly related to adipose tissue function and increased macrophage infiltration within the tissue. Macrophage accumulation is strongly correlated with adipocyte hypertrophy [8], which leads to altered adipokine secretion [9], local hypoxia [10] and adipocyte death [11]. In mice, adipose tissue macrophages (ATM) are identified via expression of F4/80 [12]. ATM include newly infiltrating macrophages (positive for CD11b and CD11c), typically surrounding necrotic adipocytes and presenting a M1 phenotype (classically activated pro-inflammatory macrophages) and resident macrophages, present in the interstitial spaces, expressing genes characteristic of M2 anti-inflammatory phenotype such as CD163 and IL-10 [13], [14], [15].

Furthermore, fat enriched diets have direct effects on the immune system even before the onset of obesity [16]. Conversely, it has been suggested that early immune challenges may predispose to subsequent body weight changes, especially when subjected to altered diets [17]. Immune cell (neutrophils, macrophages, eosinophils) infiltration changes over time (6 vs. 12 vs. 24 weeks) [18], [19] and has been shown in other tissues to be often preceded by (triggered by) complement activation. In addition, dual immune-metabolic roles for a number of innate immune complement system proteins have been demonstrated [20], [21]. In that context, C3 and its cleavage products, C3adesArg (aka acylation stimulating protein, ASP), complement C3a receptor (C3aR), tumor necrosis factor alpha (TNF-α), and toll-like receptors (TLRs), among others, are present in adipose tissue and, based primarily on mouse models, are proposed to impact adipose tissue functions, leading to insulin resistance.

C5L2 (G-protein receptor 77, GPR77) belongs to the classical chemoattractant receptor family including C5a receptor (C5aR), C3a receptor (C3aR) and formyl-Met-Leu-Phe receptor (fMLP) [22]. These receptors are activated, subsequent to immune attack, to recruit leukocytes. C5L2 is highly expressed in spleen, liver and fat depots [23], [24] and immune cells [22], [23]. C5L2 additionally plays a role in energy metabolism, as a receptor for ASP, although this remains controversial. Following binding of the ligand ASP [25], [26], [24], this stimulates fat storage [24], [27] and increases glucose transport [28]. Furthermore, higher plasma ASP levels are associated with obesity [29], cardiovascular disease [30], [31] and type 2 diabetes [32]. C5L2 also binds the ASP precursor C3a (with similar effects) as well as C5a and C5adesArg with varying affinities [33], [26], [34]. C5a, a potent anaphylatoxin, binds both C5L2 and C5aR. Binding to C5aR increases chemotaxis of leukocytes [35] while C5L2 does not [33], [34]. C5L2 is proposed as a decoy receptor, sequestering C5a and preventing binding to C5aR [33], [36], [34]. Previous studies on C5L2 deficient mice (C5L2^−/−^) have demonstrated both pro- and anti-inflammatory effects of C5L2 in situations of inflammation. Separate studies by Chen and Rittirsch point to a complex role of C5L2 in inflammation, acting as a positive modulator of C5a and C3a [37], [38]. C5a binding to C5L2 directly mediates release of the high-mobility group protein B1 (HMGB1) [39], which activates inflammatory responses [40]. Further, from a metabolic or energetic aspect, C5L2 deficient mice have altered adipose tissue function (decreased fat storage, delayed fat clearance and increased fat utilization in muscle [41].

Given the separate characteristics of the C5L2^−/−^ phenotype, the aim of the present study was to specifically examine the immune impact of C5L2^−/−^ over time under two different conditions, a routine chow diet and a moderate fat diet leading to diet-induced obesity (DIO) in various adipose depots and the impact on adipose tissue inflammation and metabolic profile.

## Materials and Methods

### Ethical statement

All animal protocols were conducted in accordance with the CACC guidelines and were approved by the Laval University animal ethics and care committees and were conducted in accordance with their guidelines, and all efforts were made to minimize suffering. All mice were housed individually in a sterile barrier facility with fade-in/fade-out 12 h light: 12 h darkness. Mice were euthanized with anesthetic overdose, followed by immediate cervical dislocation and blood and tissues were then collected at 0900 h. All efforts were made to minimize suffering.

### Mice

The C5L2 (^−/+^) heterozygous mice were initially kindly provided by Regeneron Pharmaceutics Inc. courtesy of Dr. Joseph Sorrentino (Tarrytown, NY, USA). Development of knockout mice by VelociGene is described in detail [42]. Homozygous breeding in our internal colony generated C5L2^−/−^ littermates. Wild type mice, as control (Ctl), were purchased from Charles River laboratories (St-Constant, QC, Canada) and were matched for age, sex, and initial weight with the C5L2^−/−^ cohort. There was no difference in results obtained with male and female mice, and results were pooled. All mice were housed individually in a sterile barrier facility with fade-in/fade-out 12 h light: 12 h darkness. At 8–9 weeks old, mice were placed on chow diet (11% kcal fat, 67% complex carbohydrate with no sucrose added, 21% protein at 3.4 kcal/g; Charles River Laboratories, Wilmington, PA, USA) or a moderate fat diet-induced obesity (DIO) diet (45% kcal fat, 17% complex carbohydrate, 17% sucrose, and 20% protein at 4.73 kcal/g; Research Diets Inc., New Brunswick, NJ, USA) and were euthanized at 14, 20 and 34 weeks of age after 6, 12 and 24 weeks of diet. All protocols were approved and were conducted in accordance with the CACC guidelines and approved by the Laval University Animal Care Committee. Mice were euthanized with anesthetic overdose, followed by immediate cervical dislocation and blood and tissues were then collected at 0900 h. All efforts were made to minimize suffering.

### Ex vivo assays-Primary adipocyte culture and preparation of stromal-vascular fraction (SVF)

Four white adipose tissue (WAT) depots: inguinal, pectoral, perigonadal and retroperitoneal-perirenal (retroperirenal), muscle and liver were dissected from Ctl or C5L2^−/−^ mice and weights recorded. Pectoral, retroperirenal, muscle and liver were flash frozen in liquid nitrogen and placed at −80°C for later analysis. Inguinal and perigonadal fat pads were collected under sterile conditions in Krebs Ringer buffer (KRB) and used for the evaluation of macrophage infiltration and for primary adipocyte culture. Fat pads were minced, digested with collagenase buffer (KRB + collagenase type 2 [1 mg/mL] + CaCl_2_ 0.5 mM + fatty acid free bovine serum albumin (BSA) 2% (w/v)) at 5 mL/g of tissue for 1 h at 37°C with gentle shaking. Cell homogenates were filtered through sterile nylon mesh (180 µm) and centrifuged gently at 150 g (1200 rpm) for 5 min at room temperature (RT). Adipocytes were removed from the floating layer and washed twice by centrifugation with KRB at RT. Adipocytes were incubated individually in 2 mL serum-free DMEM/F12 medium, 24 hours. After incubation, media were centrifuged (1200 rpm, 5 min) and frozen (−80°C) for later analysis.

After collagenase digestion, remaining buffer was re-suspended, filtered (70 µm), and centrifuged (600 g = 2500 rpm, 10 min, RT). The stromal-vascular fractions (SVF) were washed twice with KRB (RT), re-suspended, incubated with erythrocyte lysis buffer (K_2_HPO_4_ 5.7 mM, NH_4_Cl 155 mM, EDTA 0.1 mM, 10 min, RT), then washed twice with KRB.

### Flow cytometry analysis of macrophage composition

SVF fractions were incubated with blocking solution (0.5% bovine serum albumin in PBS, 15 min), centrifuged, re-suspended in 1^st^ antibody solution (blocking solution + rat monoclonal [BM8] to F4/80, 1 µg/mL, Abcam, Cambridge, MA, USA) and incubated (30 min, 4°C, gentle shaking). Cells were washed twice with PBS (centrifuged 5 min, 2500 rpm), incubated (30 min, 4°C) with 2^nd^ antibody (blocking solution + rabbit anti-rat IgG FITC conjugated, 6 µg/mL, Sigma, St-Louis, MO, USA). Cells were washed twice with PBS, re-suspended in 2% normal hamster serum (Cedarlane Labs, Burlington, ON, Canada) in PBS for 5 min, then incubated with 3^rd^ antibody (hamster monoclonal anti-mouse CD11c conjugated with phycoerythrin (PE), 40 µg/mL, Cedarlane Labs, Burlington, ON, Canada) for 30 min, 4°C. Finally, cells were washed twice (PBS), re-suspended in 2% paraformaldehyde (Sigma, Oakville, ON, Canada) and assayed by FACS (BD FACSCanto II, BD Biosciences, San Jose, CA, USA). WinMDI software (version 2.9, The Scripps Research Institute, Jupiter, FL, USA) was used to calculate cell distributions (%). Incubations without first and third antibodies (negative control) were used to evaluate background and set the gating for positive fluorescence. Values are expressed as percent positive gated cells. Less than 1% of the negative control cells fell above the gated setting.

### Plasma and culture media assays

Fasting plasma samples were taken by cardiac puncture. Plasma triglycerides (TG), non-esterified fatty acids (NEFA) and glucose were measured using colorimetric enzymatic kits (TG: Roche Diagnostics Indianapolis, IN; NEFA and glucose: Wako Chemicals, Richmond, VA, USA). Plasma and adipocyte cultured media were analyzed with mouse Duoset ELISA kits for adiponectin/Acrp30, CCL2/JE (MCP-1), KC/CXCL1 and complement component 5a (C5a) according to commercial assay protocols (R & D systems Inc., Minneapolis, MN, USA). Plasma insulin levels were evaluated with Ultra-Sensitive Mouse Insulin ELISA Kit (Crystal Chem Inc., Downers Grove, IL, USA) according to manufacturer's indications. Multiplex mouse cytokine profiler (Bio-plex pro™ Mouse cytokine 23-plex assay kit) was purchased from Bio-Rad (Bio-Rad Laboratories, Hercules, CA, USA) for cytokine measurements in plasma and utilised according to manufacturer specifications.

### RNA extraction and real time qPCR analysis

RNA was extracted from retroperirenal fat pads in male mice, using RNeasy Lipid Tissue Mini Kit and 0.74 µg of total RNA was retrotranscribed by RT^2^ First Strand kit (Qiagen Inc., Mississauga, ON, Canada). Genomic DNA contamination was eliminated by DNase treatment included in RT^2^ First Strand kit. Custom Mouse RT^2^ Profiler PCR Array and RT^2^ SYBR® Green qPCR Master Mix (Qiagen Inc., Mississauga, ON, Canada) was used and PCR was performed using CFX96™ Real-Time PCR Detection System (Bio-Rad Laboratories, Mississauga, ON, Canada). Specific primers for F4/80 (forward: 5′-CTTTGGCTATGGGCTTCCAGTC-3′ and reverse: 5′-GCAAGGAGGACAGAGTTTATCGTG-3′), CD11c (forward: 5′-CTGGATAGCCTTTCTTCTGCTG-3′ and reverse: 5′-GCACACTGTGTCCGAACTC-3′), CD163 (forward: 5′-GGGTCATTCAGAGGCACACTG-3′ and reverse: 5′-CTGGCTGTCCTGTCAAGGCT-3′), C5aR (forward: 5′-GGGATGTTGCAGCCCTTATCA-3′ and reverse: 5′-CGCCAGATTCAGAAACCAGATG-3′) and C3aR (forward: 5′-TAACCAGATGAGCACCACCA-3′ and reverse: 5′-TGTGAATGTTGTGTGCATGG-3′) were used. For data analysis the ΔΔCt method was used, as performed with Bio-Rad CFX manager software following the manufacturer's guidelines (Bio-Rad Laboratories, Mississauga, ON, Canada). Simultaneous normalization to three house-keeping genes (18S ribosomal RNA [18SsRNA], eukaryotic translation elongation factor 2 [Eef2] and ribosomal protein, large, P0 [Rplp0], all of which were constant, was performed. For each gene of interest, fold-changes were calculated as difference in gene expression between wild type (Ctl) mice for 6 weeks on chow diet, set at a value of 1, vs. all other conditions

### Monocyte migration assay

Bone marrow monocytes from Ctl and C5L2^−/−^ mice were isolated as follow. Bone marrow from pooled tibiae and femurs of 5 mice was extracted by gently cracking open bones and by removing bone marrow with several successive washes. Cell were filtered through 70 µm mesh nylon filters and resuspended in RoboSep Buffer + 5% rat serum (StemCell Technologies, Vancouver, Canada). The monocyte fraction was enriched using EasySep Mouse Monocyte enrichment kit following manufacturer's instructions (StemCell Technologies, Vancouver, Canada). A similar amount of bone marrow cells was used for Ctl and C5L2^−/−^ for monocyte enrichment (approximately 1.75×10^8^ cells). A similar amount of cells was counted in the monocyte enriched fraction for both strains (approximately 2×10^6^ cells). Ctl and C5L2^−/−^ adipose tissue conditioned media was generated by incubating a fixed amount of minced perigonadal adipose tissue for 5 h at 37°C in DMEM in the presence of antibiotics and antifungal reagents. Monocyte migration was assessed using QCM Chemotaxis 5 µm 24-Well Cell Migration Assay (Millipore, Billerica, MA, USA) following manufacturer's instructions. Briefly, Ctl and C5L2^−/−^ monocytes were resuspended in serum-free media and 1.66×10^5^ cells were added to each insert. Ctl or C5L2^−/−^ adipose tissue conditioned media or serum-free control was then added to the lower chambers. Plates were incubated for 20 h at 37°C in a CO_2_ incubator. Monocytes that migrated through the membrane were quantified following manufacturer's instructions.

### Statistical Analysis

Graphical results are presented as mean ± SEM for 4–18 mice per group. Groups were compared by t-tests, one-way or two-way ANOVA or by two-way repeated measures ANOVA (RM-ANOVA) followed by Bonferroni post-test using Prism 5.0 software (GraphPad Software Inc., La Jolla, CA, USA). Emphasis was placed on analysing the genotype × time factor (2-way ANOVA) rather than the diet factor. Statistical significance was set at *p*<0.05, where NS indicates not significant.

## Results

### Classical chemoattractant receptor and complement protein are modulated over time and according to dietary conditions

**Figure 1:**
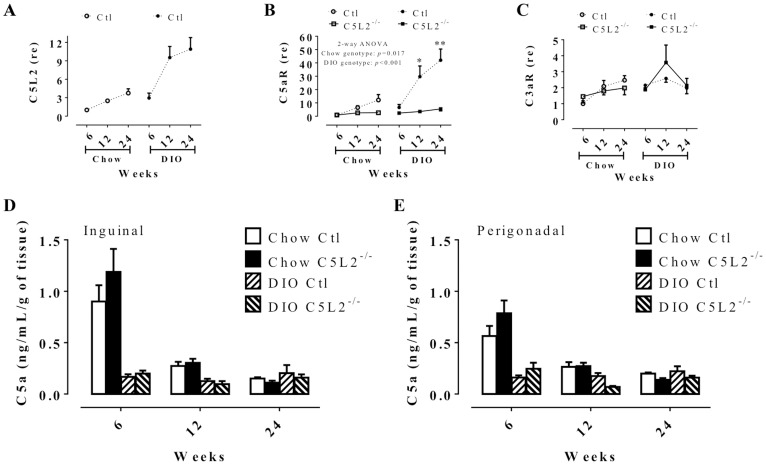
Chemoattractant receptors and related complement proteins in mouse adipose tissue. Retroperirenal adipose tissue mRNA gene expression of complement receptors C5L2 (A), C5aR (B) and C3aR (C) analysis of wild-type control (Ctl) (circle and dotted line) and C5L2^−/−^ (square and solid line) mice on chow diet (white circle or square) or diet induced obesity (DIO) (black circle or square) diet over 6, 12 and 24 weeks. The results are expressed as relative expression (re) compared to 6 weeks chow diet Ctl set as 1. Complement C5a production by primary isolated adipocytes of inguinal (D) and perigonadal (E) adipose tissue in Ctl (white bars) and C5L2^−/−^ (black bars) mice on chow diet (plain bars) or DIO (hatched bars). Data are presented as mean±SEM (n = 4–10 mice per group) with two-way (2-way) ANOVA for genotype × time between Ctl and C5L2^−/−^ for each diet with a Bonferroni post-test where * *p*<0.05 and *** *p*<0.001 for Ctl vs. C5L2^−/−^ for the same diet.

To evaluate the immune impact of total body C5L2 deletion in white adipose tissue (WAT), we first evaluated the related chemokine receptors (C5L2, C5aR and C3aR) and complement proteins which bind these receptors in WAT using two different diet conditions: a typical standard low fat complex carbohydrate chow diet, and a moderate fat-sucrose diet-induced obesity (DIO) diet with statistical emphasis on the genotype × time effects. In wild-type control (Ctl) mice, C5L2 mRNA expression is increased over time by 3-fold on chow diet (ANOVA *p* = 0.004) and by 10-fold on a diet-induced obesity diet (DIO) (ANOVA *p* = 0.017), with greater expression in DIO vs. chow diet (2 way-ANOVA, diet: *p*<0.0001) (Fig. 1A). C5aR mRNA shows a similar pattern to C5L2 for Ctl mice (Fig. 1B). However, in C5L2^−/−^ mice, C5aR mRNA is lower than in Ctl mice on both diets with little increase over time (Fig. 1B). By contrast, C3aR expression was not different between Ctl and C5L2^−/−^ mice on either diet, with no DIO or time effect (Fig. 1C). To examine if adipose tissue ligand production also changed with receptor expression, C5a and ASP/C3adesArg were measured. There was no difference in primary adipocyte C5a production between Ctl and C5L2^−/−^ mice (Fig. 1D–E), although production decreased over time on chow diet in both adipose tissues (2 way-ANOVA, inguinal; time: *p*<0.0001 and perigonadal; time: *p*<0.0001), but not on DIO (Fig. 1D–E). In these samples, ASP/C3adesArg in the media was below the detection limit.

### C5L2^−/−^ mice display increased adipose tissue macrophage content even on chow diet

**Figure 2:**
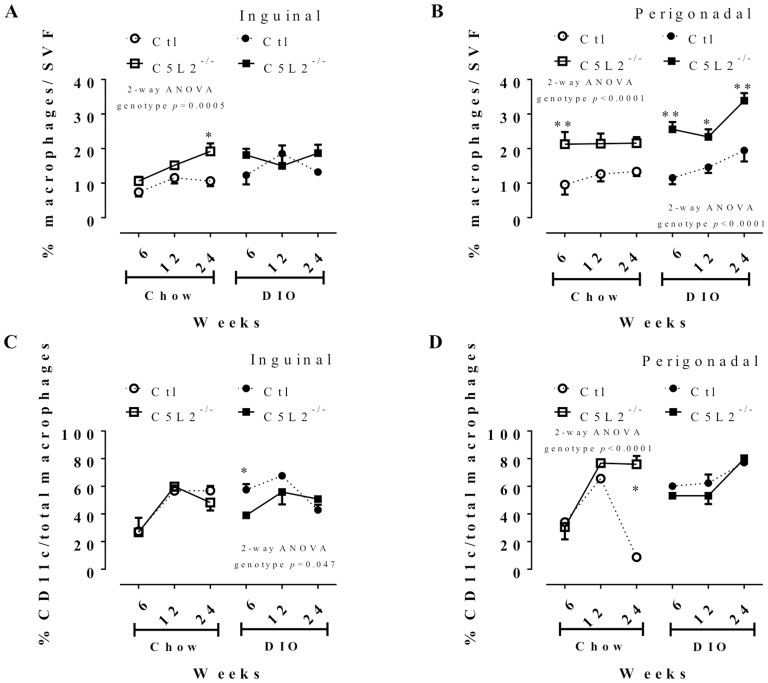
Flow cytometry analysis of F4/80 and CD11c (M1 pro-inflammatory) macrophage markers in mouse inguinal and perigonadal adipose depots. Percentage of macrophages based on F4/80 (A and B) and CD11c (C and D) markers in inguinal (A–C) and perigonadal (B–D) stromal vascular fraction (SVF) of adipose tissue after 6, 12 and 24 weeks of chow diet (white circle or square) and DIO (black circle or square) for Ctl (circle and dotted line) and C5L2^−/−^ (square and solid line) mice. Data are presented as percentage of F4/80 positive cells (total macrophage) over total cells of the SVF (A–B) or as CD11c percent of positive cells relative to total macrophages (C–D) as mean±SEM (n = 4–8 adipose tissues per group). All data were analysed with two-way (2-way) ANOVA with genotype × time between Ctl and C5L2^−/−^ for each adipose tissue with a Bonferroni post-test where * *p*<0.05 and ** *p*<0.01 for Ctl vs. C5L2^−/−^ for the same diet in the same tissue. Additional 2-way ANOVA for diet × time for % macrophages indicates a diet effect in inguinal (A) for Ctl (*p* = 0.028) and C5L2^−/−^ (*p* = NS) and in perigonadal (B) for Ctl (*p* = 0.05) and C5L2^−/−^ (*p* = 0.01).

**Figure 3:**
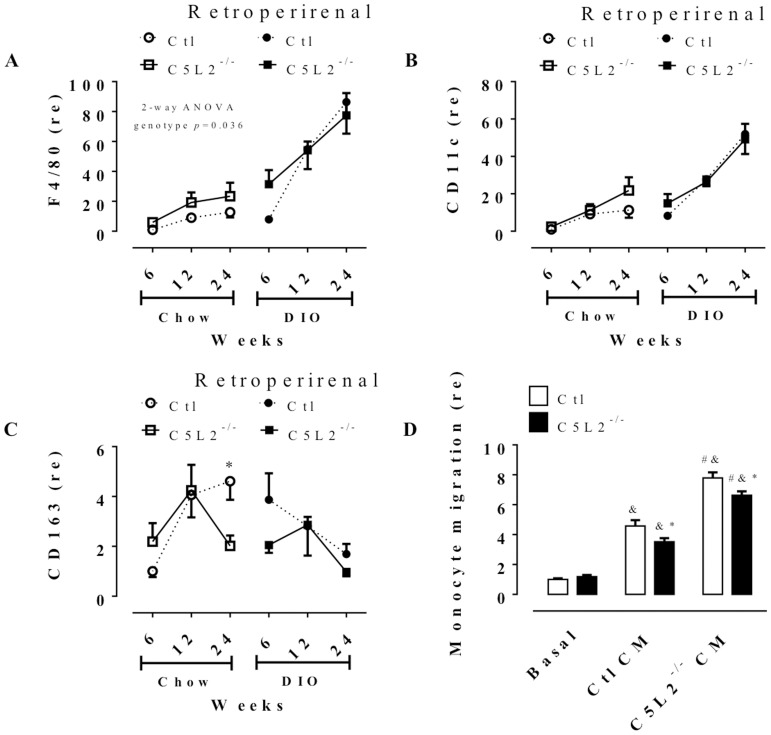
Gene expression of macrophage type distribution in mouse retroperirenal adipose depot and monocyte infiltration response to adipose tissue conditionated-media adipokine. Retroperirenal adipose tissue mRNA expressions of F4/80 (total macrophage, A), CD11c (type M1, B) and CD163 (type M2, C) in Ctl (circle and dotted line) and C5L2^−/−^ (square and solid line) on chow diet (white circle or square) or DIO (black circle of square) over 6, 12 and 24 weeks. Data are expressed as relative expression (re) mean±SEM (n = 4–5 mice per group) compared to 6 weeks, chow diet Ctl set as 1. All data were analysed with two-way (2-way) ANOVA for genotype × time between Ctl and C5L2^−/−^ for each diet or adipose tissue with a Bonferroni post-test where * *p*<0.05 and ** *p*<0.001 for Ctl vs. C5L2^−/−^ for the same diet or tissue. Monocyte migration was assessed in bone marrow monocytes isolated from Ctl and C5L2^−/−^ mice. Cells were incubated without treatment (Basal) or with conditioned media (CM) derived from gonadal adipose tissue from Ctl (Ctl CM) or C5L2^−/−^ (C5L2^−/−^ CM) mice (D). Data are expressed as relative expression (re) of monocyte migration mean±SEM (n = 3–5 migration experiments per condition). The data were analysed with regular one-way ANOVA with Bonferroni multiple comparisons test, where “&” indicates significant difference from basal condition, “#” indicates significant difference from Ctl CM and “*” indicate significant difference between the genotype of monocyte for the same condition, all *p*<0.05. Additional 2-way ANOVA for diet × time for F4/80 expression indicates a diet effect in retroperirenal (A) for Ctl (*p* = 0.0001) and C5L2^−/−^ (*p*<0.0001).

Flow cytometry analysis for cells positive for F4/80 macrophage cell marker in the stromal-vascular fraction (SVF) of two adipose fat pads (inguinal and perigonadal) was assessed at the varying diet times. C5L2^−/−^ male mice had increased % macrophage/SVF (%F4/80) content versus Ctl in inguinal and perigonadal fat on chow diet (Fig. 2A and B). Both C5L2^−/−^ and Ctl male mice have increased macrophage content with DIO challenge, with C5L2^−/−^ being further increased over Ctl in perigonadal fat. Female mice had a similar profile of macrophage content with respect to genotype (data not shown). Further, in macrophage derived from inguinal depots, there was a diet difference in Ctl (*p* = 0.028) but not in C5L2^−/−^ (*p* = NS), while there was a diet effect in macrophages from perigonadal in both Ctl (*p* = 0.05) and C5L2^−/−^ (*p* = 0.01). In retroperirenal depots, gene expression of F4/80 is higher in C5L2^−/−^ compared to Ctl on chow diet (Fig. 3A, *p* = 0.036), while there was no difference between C5L2^−/−^ and Ctl. However there was a diet effect in both Ctl (*p* = 0.0001) and C5L2^−/−^ (*p*<0.0001) mice; and an increase in both genotypes over time.

### C5L2^−/−^ mice do not have altered distribution of M1 vs M2-type macrophages

In inguinal and perigonadal fat, macrophage M1 versus M2 distribution was evaluated as CD11c cell surface positive populations relative to total macrophage content of SVF, and in retroperirenal fat, gene expression of CD11c and CD163 mRNA was evaluated. On a chow diet, M1 content is comparable between Ctl and C5L2^−/−^, (Fig. 2C, D) except for 24 weeks in perigonadal. Although total macrophage content in C5L2^−/−^ adipose tissue increased on DIO, the M1 vs M2 distribution is comparable to Ctl mice (Fig. 2C, D). This is also reflected in CD11c mRNA, which increases with time and DIO in both genotypes similarly (Fig. 3B). M2-marker CD163 mRNA expression was comparable between genotypes on chow diet and DIO, except at 24 weeks on chow diet. This increase in M2 CD163 expression in retroperirenal is consistent with the decrease in % CD11c in perigonadal (Fig. 3C).

### C5L2^−/−^ secreted adipokines are more chemoattractant for monocytes

In order to determine why C5L2^−/−^ adipose tissue had more macrophage presence than Ctl adipose tissue without a significant difference in macrophage distribution, a monocyte-migration experiment was performed (Fig. 3D). In the absence of stimulation (basal conditions) Ctl and C5L2^−/−^ monocytes collected from bone marrow have equivalent monocyte migration capacity. When cells are incubated in the presence of conditioned-media from Ctl adipose tissue, cell migration increased in both Ctl and C5L2^−/−^ cells (≈ 4-fold) compare to the basal (non-stimulated) state, with the C5L2^−/−^ monocytes migrating less than the Ctl cells. Moreover, when monocytes were incubated in the presence of conditioned-media derived from C5L2^−/−^ adipose tissue there was a greater migration capacity of both Ctl and C5L2^−/−^ cells (≈7-fold), but again with C5L2^−/−^ monocytes demonstrating less migration (Fig. 3D).

### C5L2^−/−^ mice have altered circulating and adipose tissue inflammatory-related cytokines

**Figure 4:**
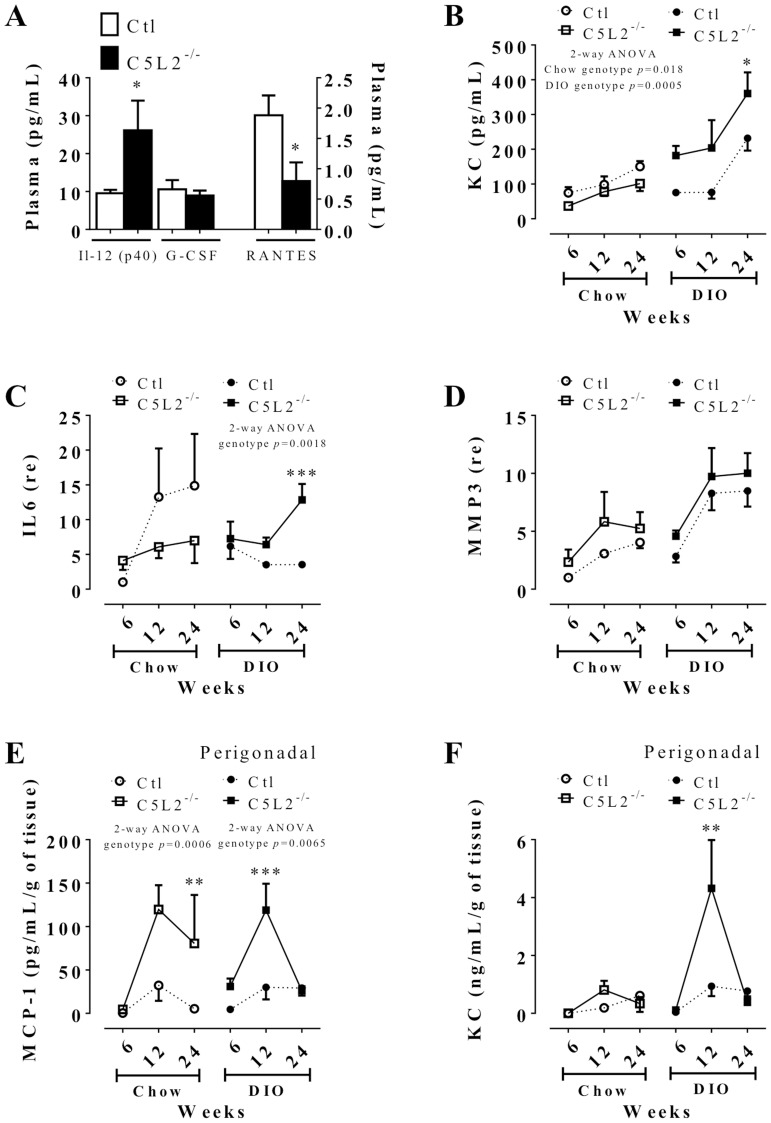
Systemic and adipose tissue cytokine profile in mice. Mouse cytokine profiler analysis in pooled plasma: (A) Data are expressed as mean±SEM of n = 8 Ctl and C5L2^−/−^ mice for each time point and for each diet. (B) Plasma keratinocyte derived chemokine (KC) analysis of Ctl (circle and dotted line) and C5L2^−/−^ (square and solid line) of mice on chow diet (white circle or square) or DIO (black circle or square) over 6, 12 and 24 weeks. Retroperirenal adipose tissue mRNA expression of interleukin 6 (IL-6) (C) and matrix metalloproteinase 3 (MMP3) (D) in Ctl (circle and dotted line) and C5L2^−/−^ (square solid line) mice on chow diet (white circle or square) or DIO (black circle or square) over 6, 12 and 24 weeks. Data are expressed as relative expression (re) mean±SEM (n = 4–5 mice per group) compared to 6 weeks chow diet Ctl set at 1. Evaluation of monocyte chemoattractant protein-1 (MCP-1) (E) and KC (F) in adipocyte primary cell culture media for 24 h isolated from perigonadal adipose tissue after 6, 12 and 24 weeks on chow diet (white circle or square) and DIO (black circle or square) of Ctl (circle and dotted line) and C5L2^−/−^ mice (square and solid line). Data are presented as means±SEM (3–10 adipose tissue media per group) over time and expressed as pg/mL or ng/mL per gram (g) of tissue used. Paired t-test for each plasma cytokine value where * *p*<0.05 for C5L2^−/−^ vs. Ctl (A) and two-way (2-way) ANOVA for genotype × time between Ctl and C5L2^−/−^ for each diet or adipose tissue or media with a Bonferroni post-test where * *p*<0.05 and ** *p*<0.01 and *** *p*<0.001 for C5L2^−/−^ vs. Ctl on same diet or adipose tissue or media. Abbreviations: Il-12(p40), subunit beta of interleukin 12; G-CSF, granulocyte colony stimulating factor; RANTES, Regulated upon Activation, Normal T-cell Expressed and Secreted or CCL5.

Cytokine levels were evaluated by mouse multiplex profiler on pooled plasma. Only interleukin 12 (Il-12β (p40)), granulocyte colony stimulating factor (G-CSF) and Regulated upon Activation, Normal T-cell Expressed, and Secreted (RANTES or CCL5) were at detectable levels at all the different times, diets and genotype groups. As shown in Fig. 4A, Il-12(p40) was increased and RANTES was decreased, while there was no change in G-CSF in C5L2^−/−^ mice plasma vs. Ctl mice. Keratinocyte derived chemokine (KC), the ortholog of IL-8 in humans, measured individually, was increased in C5L2^−/−^ mice, over time and with DIO challenge (Fig. 4B). Although there was increased macrophage content in adipose tissue, mRNA expression of TNF-α and nuclear factor kappa-light-chain-enhancer of activated B cells (NFκB1) were not affected by genotype, time or diet (data not shown). On the other hand, interleukin 6 (IL-6) was elevated in C5L2^−/−^ on DIO relative to Ctl (Fig. 4C). Additionally, matrix metalloproteinase 3 (MMP3), implicated in tissue remodeling, increased with time and diet but not genotype on both diets (Fig. 4D).

To evaluate the potential influence of adipose cytokine secretions on macrophage recruitment, monocyte chemoattractant protein 1 (MCP-1), C5a and KC production by primary adipocytes were evaluated. MCP-1 secretion was higher in C5L2^−/−^ primary cultured adipocytes on both diets, especially at 12 weeks (Fig. 4E). KC adipocyte secretion was also modulated over time, with a genotype difference at 12 weeks on DIO regimen (Fig. 4F). Production of MCP-1 and KC by inguinal adipocytes also showed the same general pattern as with perigonadal adipocytes; although the secretion levels were lower (data not shown).

### C5L2^−/−^ mice have higher delta body weight, food intake and white adipose tissue weight compared to the control group on both diets

C5L2^−/−^ mice on both diets (chow and DIO) had greater body weight gain (delta body weight, Table S1 and Fig. S1A), in spite of being age, gender and weight matched at the beginning of the studies, due to increased WAT (inguinal, perigonadal, pectoral and retroperirenal, Fig. S1B). These results are at least in part explainable by the increased cumulative food intake over the time period for C5L2^−/−^ mice, again on both chow diet and DIO (Fig. S1C). In addition, the DIO treated mice (both C5L2^−/−^ and Ctl) had greater weight gain on the DIO compared to the chow diet (Figure S1A), with a greater total WAT size (Figure S1B) for both Ctl (*p* = 0.005) and C5L2^−/−^ (*p*<0.0001) mice.

### C5L2^−/−^ have altered expression of lipid-related genes in adipose tissue compared to Ctl mice

**Figure 5:**
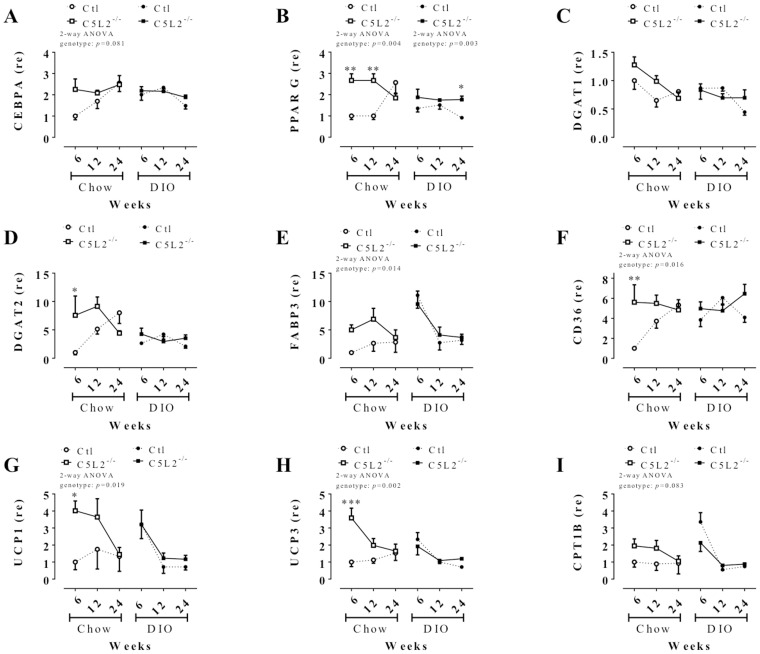
Adipose tissue lipid metabolism gene expression. Retroperirenal adipose tissue mRNA gene expression for genes involved in lipid metabolism in Ctl (circle and dotted line) and C5L2^−/−^ (square and solid line) mice on chow diet (white circle or square) and DIO (black circle or square). Adipogenesis-related genes (A–B); triglyceride synthesis-related genes (C–D); cell membrane fatty acid transport-related genes (E–F); thermogenesis-related genes (G–H) and mitochondrial long chain fatty acid transport-related genes (I). Data are expressed as relative expression (re) mean±SEM (n = 4–5 mice per group) compared to 6 weeks chow diet Ctl set as 1 where two-way (2-way) ANOVA for genotype × time between Ctl and C5L2^−/−^ for each diet or adipose tissue with a Bonferroni post-test where * *p*<0.05, ** *p*<0.01 and *** *p*<0.001 for C5L2^−/−^ vs. Ctl. Abbreviations: CEBPA, CCAAT/enhancer-binding protein α; PPARG, peroxisome proliferator-activated receptor γ; DGAT1 and 2, diacylglycerol O- acyltransferase 1 and 2; FABP3, fatty acid binding protein 3; CD36 or FAT, fatty acid transporter; UCP1 and 3, uncoupling protein 1 and 3; CPT1B, carnitine palmitoyltransferase 1B.

Local (adipose tissue) and systemic (plasma) lipid parameters and gene expression in retroperirenal adipose tissue were evaluated in C5L2^−/−^ vs. Ctl mice. On a chow diet, plasma triglyceride was decreased in C5L2^−/−^ vs. Ctl, while both triglyceride and NEFA levels were decreased in C5L2^−/−^ vs. Ctl mice with DIO challenge (Table S1). Gene expression in adipose tissue was evaluated on both chow diet and DIO. On chow diet, mRNA expression of a number of lipid metabolism-related genes were different between C5L2^−/−^ and Ctl mice, especially at 6 weeks (Fig. 5). Specifically, the transcription factor CCAAT/enhancer-binding protein α (CEBPA) and the nuclear hormone receptor peroxisome proliferator-activated receptor γ (PPARG) which contribute to adipogenesis [43] were higher in C5L2^−/−^ mice (Fig. 5A–B). Diacylglycerol-O-acyltransferase 1 and 2 (DGAT1 and 2) genes, enzymes exclusive for triglyceride synthesis, tended to be higher in C5L2^−/−^ mice (Fig. 5C–D). Genes involved in fatty acid metabolism, fatty acid binding protein 3 (FABP3) and CD36 (aka fatty acid transporter, FAT) were also higher in C5L2^−/−^ mice (Fig. 5E–F). Uncoupling protein 1 and 3 (UCP1 and 3), implicated in mitochondrial thermogenesis [44], and carnitine palmitoyltransferase 1B (CPT1B), required for transport of long-chain fatty acid into mitochondria [45], were higher in C5L2^−/−^ mice at 6 weeks, decreasing gradually to reach Ctl levels at 24 weeks (Fig. 5G–H–I). By contrast, with DIO challenge, while there were time related changes in gene expression, there was no difference in C5L2^−/−^ mice vs. Ctl mice, excepted for PPARG.

### C5L2^−/−^ mice have altered glucose and insulin metabolism with a DIO challenge

**Figure 6:**
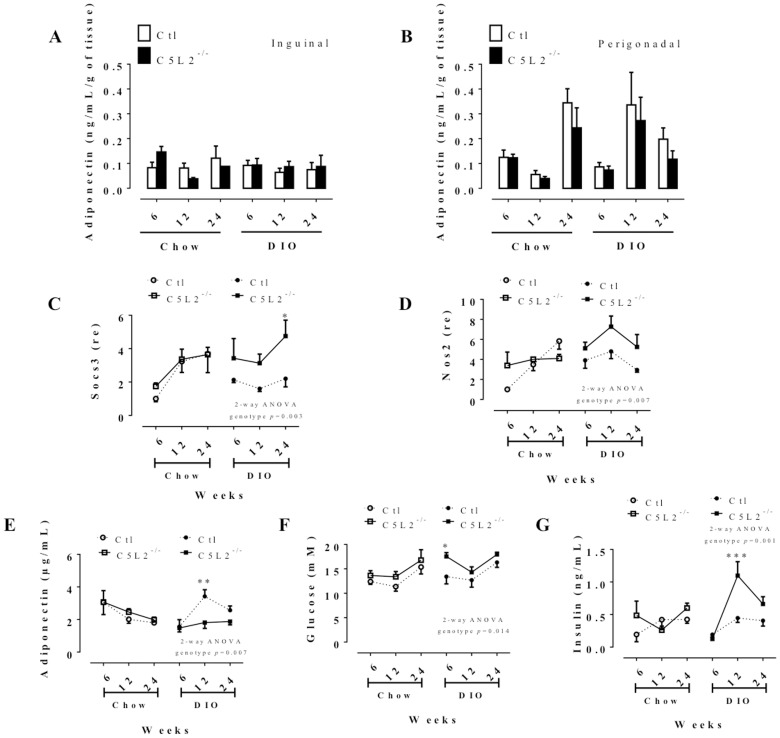
Insulin metabolism and insulin resistance markers in mice. Evaluation of adiponectin in Ctl (white bars) and C5L2^−/−^ (black bars) adipocyte primary cell culture media for 24 h isolated from inguinal (A) and perigonadal (B) adipose tissue after 6, 12 and 24 weeks on chow diet and DIO. Data are presented as means±SEM (3–10 adipose tissue media per group) over time and expressed as pg/mL or ng/mL per gram (g) of tissue used. Retroperirenal adipose tissue mRNA gene expression for genes related to insulin resistance (C–D) from Ctl (circle and dotted line) and C5L2^−/−^ (square and solid line) mice on chow diet (white circle or square) and DIO (black circle or square). Data are expressed as relative expression (re) mean±SEM (n = 4–5 mice per group) compared to 6 weeks chow diet Ctl set as 1. Plasma levels of adiponectin (E), glucose (F) and insulin in Ctl (circle and dotted line) and C5L2^−/−^ (square and solid line) mice on chow diet (white circle or square) and DIO (black circle or square). Data are presented as mean±SEM (n = 8–10 mice per group) over time with two-way (2-way) ANOVA for genotype × time between Ctl and C5L2^−/−^ for each diet or adipose tissue with a Bonferroni post-test where * *p*<0.05, ** *p*<0.01 and *** *p*<0.001 for C5L2^−/−^ vs. Ctl. Abbreviations: SOCS3, suppressor of cytokine signaling 3; NOS2, nitric oxide synthase 2.

In spite of the altered macrophage content and changes in pro-inflammatory cytokines, there was no significant genotype influence on adiponectin secretion in either inguinal or perigonadal adipocytes, although levels tended to be lower in perigonadal C5L2^−/−^ mice (Fig. 6A–B). Adiponectin, which is an insulin sensitizing agent, is involved in glucose metabolism and correlates positively with insulin sensitivity [46]. Further, mRNA expression of insulin pathway-related genes was evaluated in retroperirenal WAT over time and on both diets. Although DIO induced decreases in insulin receptor substrate 1 (IRS1) and glucose transporter type 4 (SIC2A4), there were no differences between C5L2^−/−^ and Ctl mice. There were also no genotype-related differences for protein kinase B (PKB)/AKT mRNA (data not shown). Genes typically associated with insulin resistance, tumor necrosis factor α (TNF-α) and Signal transducer and activator of transcription 3 (STAT3), likewise did not show differences between genotypes at all times points (data not shown), in spite of the increased macrophage content in C5L2^−/−^. However, on DIO, expression of Suppressor of cytokine signaling 3 (SOCS3) and nitric oxide synthase 2 (NOS2) were significantly increased in C5L2^−/−^ mice at all time points (Fig. 6C–D). While there was no difference on the chow regimen, DIO resulted in decreased adiponectin (Fig. 6E), increased glucose (Fig. 6F) and increased insulin (Fig. 6G) levels in C5L2^−/−^ mice plasma, although there was no significant diet effect on plasma glucose or insulin between genotype, likely related to the moderate fat content of the DIO diet used.

## Discussion

Innate immune systems, especially the complement system, have been proposed to play a dual role in biology of WAT [21], [20]. One well known example is that of C3a vs. ASP/C3adesArg where a minimal structural change determines if the molecule plays a role as an immune modulator or as a metabolic mediator. C3a is a powerful anaphylatoxin, which, upon binding to its receptor C3aR, enhances chemotaxis of immune cells to sites of inflammation. Cleavage of the carboxyl terminal arginine of C3a by carboxypeptidase, produces C3adesArg (aka ASP), which loses the capacity to bind to C3aR, but, through binding to C5L2 receptor, stimulates triglyceride synthesis and glucose transport in adipocytes. Moreover, classical adipokines such as leptin, adiponectin, resistin and visfatin not only affect metabolic functions but also act as potent pro- or anti-inflammatory modulators. The duality of these adipokines/cytokines provides a bridge between metabolism and inflammation and could act as triggers for the obesity-associated pathologies of insulin resistance and atherosclerosis.

C5L2, proposed as a receptor for ASP, also binds C5a and C5adesArg, and in this context, has been evaluated for its pro- and anti-inflammatory impact primarily in inflammatory situations [33]–[39]. Our study demonstrates that a deletion of C5L2 receptor has a considerable impact on both energy metabolism and the immune environment of WAT in mice with repercussions at the systemic level. Moreover, in the obese state, the impact leads to a worsening metabolic state. The other chemoattractant receptors related to C5L2, C5aR and C3aR, are also expressed in WAT and show altered expression pattern in the absence of C5L2 on DIO. C3aR adipose tissue expression is not affected by C5L2 deletion, but is higher with a DIO challenge, consistent with a previous study [47]. By contrast, C5aR adipose tissue expression increases with age and is markedly increased with a DIO challenge in wild-type Ctl mice. However, in C5L2^−/−^ mice, C5aR mRNA adipose tissue expression is reduced compared to wild-type Ctl mice. We speculate that a lack of C5L2, which is proposed as a decoy receptor for C5a, might increase binding of C5a to its receptor C5aR, leading to internalization, and contributing to a retroinhibition of the receptor gene expression, without changing the adipocyte production of C5a, which was similar between wild-type Ctl and C5L2^−/−^ mice.

As with C3a–C3aR interaction, binding of C5a to C5aR enhances chemotaxis of immune cells to the site of inflammation. The greater bioavailability of C5a to act through its receptor C5aR, could explain the increased macrophage presence seen in C5L2^−/−^ WAT mice, which was documented. In spite of increased macrophage content, the relative distribution between M1 pro-inflammatory infiltrating macrophages and M2 anti-inflammatory resident macrophages was generally unchanged. While the relative macrophage content in WAT seems to be closely regulated, and is affected by age, diet type and time on diet, genotype (presence/absence of C5L2) was also predictive of macrophage infiltration within the adipose tissue. Outside of direct WAT effects, administration of C5a centrally in the brain stimulates food intake in mice [48], [49], purportedly via interaction with C5aR. Thus, in the absence of the C5a decoy receptor C5L2, C5L2^−/−^ mice have increased food intake, which could in turn contribute to increased WAT weight and, ultimately, higher macrophage presence in WAT. Our previous study on C5L2^−/−^ mice [41] also documented increased food intake, although in spite of this, the body weights remained comparable to wild-type Ctl mice.

Interestingly, in the C5L2 deficient mice, a higher incidence of macrophages in WAT, and altered adipocyte cytokine production are present even in the chow diet regimen. Moreover, these mice demonstrate an altered WAT lipid metabolism, especially at an early age on the regular chow diet; with aging, the characteristics of C5L2^−/−^ WAT become more similar to wild-type Ctl mice. Previous studies with C5L2^−/−^ mice on chow diet showed altered lipid clearance, with decreased triglyceride synthesis capacity in WAT, although on average adipocytes had the same cell volume and triglyceride content [41]. Therefore, the increased macrophage content would not appear to be a consequence of adipocyte hypertrophy, which is known to contribute to both macrophage infiltration into WAT [8] and altered adipocyte cytokine production [9]. On the other hand, MCP-1 and KC are known to play a role in macrophage infiltration [50], [51]. With increased production of MCP-1 on both chow diet and DIO and KC on DIO by perigonadal adipose tissue, this could be another explanation of the increase presence of macrophages.

With a higher glucose and insulin plasma level; higher mRNA expression of insulin resistance-related genes in WAT and the onset of inflammation, all of which are characteristic of an insulin resistance state, C5L2^−/−^ mice have a phenotype of early insulin-resistant state compared to the wild-type Ctl mice. This is in accordance with the study by Fisette et al. who demonstrated that C5L2^−/−^ versus wild-type mice, challenged with a diabetogenic diet, develop a worsening insulin resistance state mediated through a pro-inflammatory phenotype, an altered substrate partitioning and ectopic fat deposition [52]. However, which metabolic-immune changes identified are a consequence of absence of ASP-C5L2 or absence of C5a-C5L2 interaction, or an enhancement of C5a-C5aR interaction remains to be determined.

Nonetheless, as the complement pathway is one of the first immune systems activated in response to inflammation, and complement proteins appears to play a dual role in adipose tissue, understanding the metabolism-immunity crosstalk of the complement pathway in WAT may identify strategies and therapeutic avenues for targeting insulin resistance and/or type 2 diabetes.

## Supporting Information

Figure S1 Body weight, white adipose tissue (WAT) weight and cumulative food consumption of mice.Delta body weight curve over 24 weeks (A), sum of four different fat pads (inguinal, perigonadal, pectoral and retroperirenal) at 6, 12 and 24 weeks (B) and cumulative food intake curve over 24 weeks (C) of Ctl (circle and dotted line) and C5L2^−/−^ (square and solid line) on Chow (white circle or square) or DIO (black circle or square) diet. Data are expressed as mean±SEM (n = 8–18 mice per group). Two-way ANOVA for genotype × time between Ctl and C5L2^−/−^ for each diet with a Bonferroni post-test where * *p*<0.05, ** *p*<0.01 and *** *p*<0.001 for C5L2^−/−^ vs. Ctl for the same diet. Additional 2-way ANOVA for diet × time indicates a significant diet effect on total WAT accumulation (B) for Ctl (*p* = 0.005) and C5L2^−/−^ (*p*<0.0001).
(TIF)

Table S1 Anthropometric measures and fasting plasma values.
(DOC)
