# Supplementary figures and images for: Deficiency of C5L2 Increases Macrophage Infiltration and Alters Adipose Tissue Function in Mice
Source: PLoS One. 2013 Apr 22;8(4):e60795. doi: 10.1371/journal.pone.0060795 (PMC3632610; doi:10.1371/journal.pone.0060795)

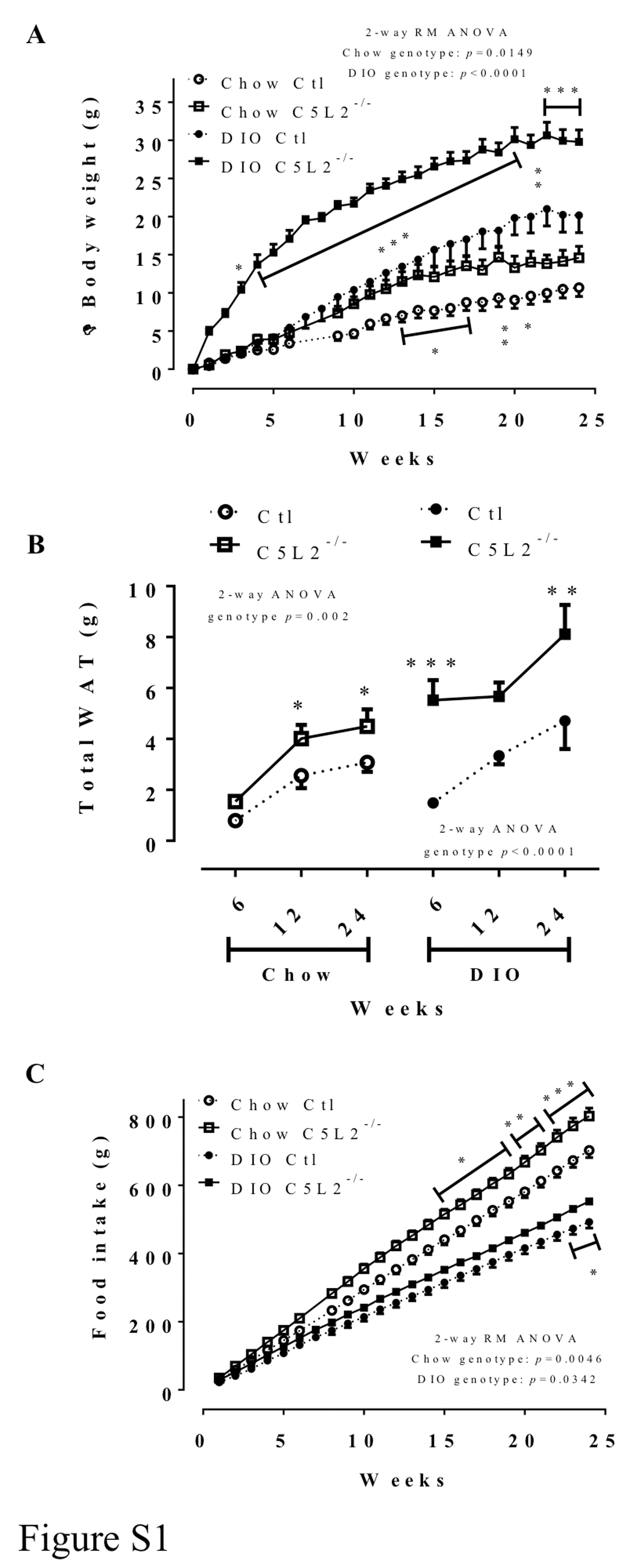

Supplement: Figure S1 — Body weight, white adipose tissue (WAT) weight and cumulative food consumption of mice.Delta body weight curve over 24 weeks (A), sum of four different fat pads (inguinal, perigonadal, pectoral and retroperirenal) at 6, 12 and 24 weeks (B) and cumulative food intake curve over 24 weeks (C) of Ctl (circle and dotted line) and C5L2−/− (square and solid line) on Chow (white circle or square) or DIO (black circle or square) diet. Data are expressed as mean±SEM (n = 8–18 mice per group). Two-way ANOVA for genotype × time between Ctl and C5L2−/− for each diet with a Bonferroni post-test where * p<0.05, ** p<0.01 and *** p<0.001 for C5L2−/− vs. Ctl for the same diet. Additional 2-way ANOVA for diet × time indicates a significant diet effect on total WAT accumulation (B) for Ctl (p = 0.005) and C5L2−/− (p<0.0001). (TIF) [file pone.0060795.s001.tif]
