# Supplementary material for: Deficiency of C5L2 Increases Macrophage Infiltration and Alters Adipose Tissue Function in Mice
Source: PLoS One. 2013 Apr 22;8(4):e60795. doi: 10.1371/journal.pone.0060795 (PMC3632610; doi:10.1371/journal.pone.0060795)
Supplement: Table S1 — Anthropometric measures and fasting plasma values. (DOC) [file pone.0060795.s002.doc]

**Table S1:** Anthropometric measures and fasting plasma values

|  | Chow diet | | | | | | 2-way  ANOVA  *p* | Diet induced obesity | | | | | | 2-way  ANOVA  *p* |
| --- | --- | --- | --- | --- | --- | --- | --- | --- | --- | --- | --- | --- | --- | --- |
|  | 6 weeks | | 12 weeks | | 24 weeks | |  | 6 weeks | | 12 weeks | | 24 weeks | |  |
|  | Ctl | C5L2^-/-^ | Ctl | C5L2^-/-^ | Ctl | C5L2^-/-^ |  | Ctl | C5L2^-/-^ | Ctl | C5L2^-/-^ | Ctl | C5L2^-/-^ |  |
| Δ Body weight (g) | 3.3  ±0.4 | 6.1  ±0.5* | 7.3  ±0.9 | 9.7  ±1.2 | 10.1  ±1.1 | 14.3  ±1.4** | <0.0001 | 6.7  ±1.6 | 9.6  ±1.7 | 12.5  ±1.1 | 19.0  ±1.5* | 20.4  ±2.4 | 30.2  ±1.3*** | <0.0001 |
| TG  (mmol/L) | 0.42  ±0.06 | 0.21  ±0.03* | 0.48  ±0.06 | 0.32  ±0.04 | 0.55  ±0.06 | 0.53  ±0.04 | 0.0035 | 0.27  ±0.04 | 0.21  ±0.03 | 0.34  ±0.04 | 0.18  ±0.02* | 0.51  ±0.05 | 0.37  ±0.05 | 0.0009 |
| NEFA (mmol/L) | 0.36  ±0.04 | 0.39  ±0.05 | 0.38  ±0.05 | 0.34  ±0.03 | 0.47  ±0.06 | 0.51  ±0.05 | 0.7854 | 0.47  ±0.06 | 0.39  ±0.06 | 0.78  ±0.11 | 0.44  ±0.12* | 0.60  ±0.08 | 0.44  ±0.05 | 0.0080 |

Values are presented as mean±SEM (n=8-10 mice per group) with two-way (2-way) ANOVA over time between Ctl and C5L2^-/-^ for each measurement with Bonferroni post-test where * *p*<0.05, ** *p*< 0.01 and *** *p*<0.001 for C5L2^-/-^ vs. Ctl at the same time.
